# Dictionary of Practical Surgery

**Published:** 1886-12

**Authors:** 


DICTIONARY OF PRACTICAL SURGERY. *By Various Hospital Surgeons. Edited by* CHRISTOPHER HEATH, F.R.C.S., *Holme Professor of Clinical Surgery in University College, etc., etc.; pp. 1854. Philadelphia:* J. B. LIPPINCOTT COMPANY, 1886.

This book is apparently intended to stand in the same relation to the usual treatise on the principles and practice of surgery in which Quain's Dictionary of Medicine stands to the usual so-called "Practice of Medicine." The editor says that it is intended for the use of men who are too busy to investigate thoroughly the subjects upon which they want information. The surgical affections are described in alphabetical order, except such as it has been found more convenient to group together in a series, as *e.g.*, those of the breast. Abundant cross-references are given. The subjects are treated of in the following order: 1, Cause; 2, Pathology; 3, Symptoms and Diagnosis; 4, Treatment; 5, Prognosis. Each writer has signed his articles and has thus been made responsible for his own statements. The editor has exercised only a general supervision, and has endeavored to exclude crude theories and doubtful practices. There are no illustrations. "The aim of the editor has been to produce a compendium of the practice of British surgery of the present day," and as none of the articles are more than two years old, he thinks that aim has been fairly attained. In special fields of surgery, as that of the abdomen, for example, the book cannot serve as a complete guide, but for the purpose for which it is intended it is reliable and quite complete. One feature which deserves special mention and commendation is, that many slight ailments which the usual treatise does not mention, but of which a physician frequently wants some additional and reliable information, are carefully and tersely described. The articles on the various skin diseases are a desirable feature and are well written. The book as a whole is to be strongly commended. E. W.

